# SETBP1 mutation determines sensitivity to immune checkpoint inhibitors in melanoma and NSCLC

**DOI:** 10.18632/aging.204913

**Published:** 2023-08-02

**Authors:** Fengxiao An, Wenjing Zhang, Yuxian Guo, Fuyan Shi, Yujia Kong, Liguo Tang, Caijing Han, Qinghua Wang

**Affiliations:** 1 Department of Clinical Laboratory, Affiliated Hospital of Weifang Medical University, Weifang 261031, Shandong, China; 2 Department of Health Statistics, Key Laboratory of Medicine and Health of Shandong Province, School of Public Health, Weifang Medical University, Weifang 261053, Shandong, China; 3 Department of Orthopedics, Sunshine Union Hospital, Weifang 261061, Shandong, China; 4 School of Public Health, Weifang Medical University, Weifang 261053, Shandong, China

**Keywords:** SETBP1 mutation, ICI treatment, melanoma, NSCLC, clinical biomarker

## Abstract

SET binding protein 1 (SETBP1) plays crucial roles in various biological processes; however, its involvement in cancer immune checkpoint inhibitor (ICI) treatments has never been studied. In this study, we collected a total of 631 melanoma and 109 non-small cell lung cancer (NSCLC) samples treated with ICI agents (i.e., anti-CTLA-4, anti-PD-1/PD-L1, or combination therapy). Additionally, we obtained their corresponding somatic mutational profiles. We observed that SETBP1 mutated (SETBP1-MUT) melanoma patients exhibited significantly prolonged ICI survival outcomes compared to wild-type patients (HR: 0.56, 95% CI: 0.38-0.81, *P* = 0.002). Consistently, an elevated ICI response rate was also noticed in the SETBP1-MUT group (42.9% vs. 29.1%, *P* = 0.016). The Association of SETBP1 mutations with favorable immunotherapeutic prognosis and response was further supported by an independent NSCLC cohort (both *P* < 0.05). Additional immunological analyses revealed that favorable immune infiltration, tumor immunogenicity, and immune response circuits were enriched in SETBP1-MUT patients. Overall, our findings suggest that SETBP1 mutations may serve as a new biomarker for stratifying beneficiaries of ICI treatments in melanoma and NSCLC, which provides possible evidence for tailoring clinical immunotherapeutic strategies.

## INTRODUCTION

Cancer patients often have a dysfunctional immune system, which can be targeted through the use of distinct immune checkpoint inhibitors (ICI) [1]. ICI treatments have produced remarkable clinical effects and significantly extended survival outcomes for many cancer types, such as melanoma and non-small cell lung cancer (NSCLC) [1]. Although melanoma and NSCLC have different pathological and clinical characteristics. However, due to the widespread use of ICI treatments in these two cancer types and the availability of numerous publicly accessible datasets, they are always selected for immune-related research. The main objective of ICI treatment is to kill cancer cells by reactivating CD8 T cell-mediated immune functions [2]. However, despite the remarkable clinical treatment efficacy, only a smaller proportion of cancer patients are responsive to ICI agents, and highly sensitive indicators of response to such treatments are not yet utilized to evaluate clinical immunotherapeutic benefits [1, 3]. Therefore, there is a need to identify new and robust indicators for predicting ICI response and selecting patients who will benefit from such treatments.

Previous evidence has demonstrated the critical roles that mutations in a single gene can play in cancer progression, immune regulation, and immune treatment response. For example, Feng et al. curated comprehensive somatic mutational profiles and clinical immunotherapy information from NSCLC patients and found that FAT1 mutations were predictive of favorable tumor immune infiltration and, importantly, better ICI treatment efficacy [4]. Consistently, the above observations were confirmed by a recent study on NSCLC patients [5]. Additionally, Zhang et al. observed that FAT1 mutations were linked to preferable ICI efficacy and immunogenicity in 109 NSCLC patients and validated these connections in 631 melanoma and 1661 pan-cancer patients [6]. Furthermore, mutations in MUC16 [7], TP53 [8], COL3A1 [9], HSPG2 [2], POLE [10], PTPRT [11], and PPP6C [12] have been shown to positively connect with ICI therapeutic efficacy. Nevertheless, JAK1/2 [13] and B2M [13] mutations were found to be negatively associated with treatment response.

SET binding protein 1 (SETBP1) is an important transcription factor that plays vital roles in multiple biological processes, such as DNA replication. Several previous studies have revealed that SETBP1 drives potential molecular mechanisms in hematologic malignancies. For example, Pacharne et al. reported that the upregulation of SETBP1 promoted FLT3-mutated acute myeloid leukemia [14]. Mutant SETBP1 enhanced the activity of the MYC pathway to facilitate CSF3R-related myeloproliferative neoplasms [15], and the NRAS-driven MAPK signal was activated by mutated SETBP1 to accelerate aggressive leukemia [16]. In patients with myelodysplastic syndrome, SETBP1 mutations were identified as predicting a poorer survival outcome [17]. SETBP1 is also involved in other cancer types, such as ovarian and gastric cancers. For instance, a study showed that the SETBP1 pathway was regulated by TRIM29 to promote the progression of ovarian cancer [18], and SETBP1 overexpression acted as a poor prognosticator in gastric cancer [19]. Recent evidence has shown that SETBP1 mediates immune regulation and anti-tumor immune infiltration [20]. However, to our knowledge, the clinical significance of SETBP1 mutations in cancer ICI treatments has not been elucidated.

Cancer immunotherapy is commonly used for two cancer types of melanoma and NSCLC. In this integrated study, we collected genomic mutational data and clinical immune treatment information for both tumor types and performed multi-dimensional immunological analyses. Our findings suggest that SETBP1 mutations could be a promising biomarker in clinical cancer immunotherapies.

## RESULTS

### SETBP1 mutations in melanoma

**Figure 1:**
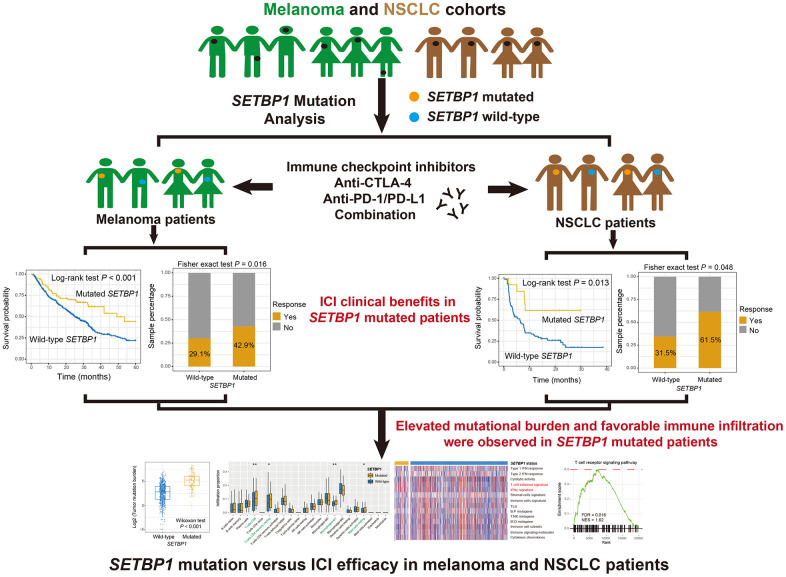
**The detailed work process of this study.** SETBP1 mutations were determined as a potential biomarker for melanoma and NSCLC clinical ICI treatments.

The detailed work process of this study was shown in Figure 1. Among the aggregated 631 melanoma samples, C > T substitution was the main mutational feature (Supplementary Figure 1). A waterfall plot was finished to show the concrete mutational pattern of SETBP1 mutations and their association with melanoma’s other driver genes and clinical factors (Supplementary Figure 1). A total of 84 (13.3%) of 631 melanoma patients harbored SETBP1 mutations and SETBP1 mutation-produced changes in the amino acid level were illustrated in Supplementary Figure 2.

### SETBP1 mutations determined the sensitivity to ICI treatments in melanoma

**Figure 2:**
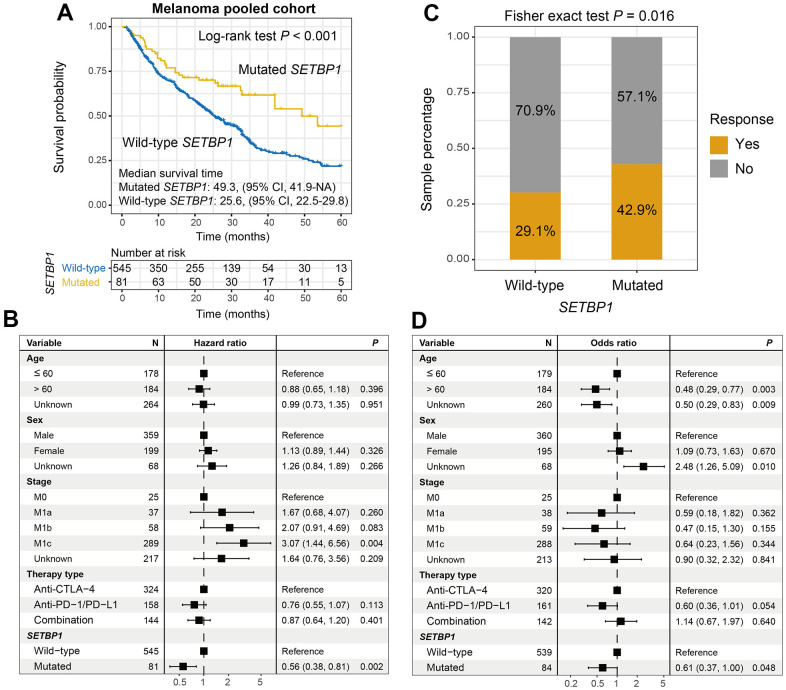
**SETBP1 mutations determined the sensitivity to ICI treatments in melanoma.** (**A**) ICI survival differences between SETBP1 mutated and wild-type subgroups. (**B**) A multivariate Cox regression analysis was performed to verify the connection between SETBP1 mutations and ICI prognosis. (**C**) ICI response rate differences of SETBP1 two subgroups. (**D**) A multivariate logistic regression analysis was performed to verify the connection between SETBP1 mutations and ICI response rate.

We first explored the ICI prognostic roles of SETBP1 mutations in pooled melanoma patients. Kaplan-Meier results showed that SETBP1 mutated (SETBP1-MUT) melanoma patients exhibited a markedly improved ICI survival benefit than wild-type patients (median survival time: 49.3 vs. 25.6 months, Log-rank test *P* < 0.001; Figure 2A). A multivariate Cox regression model of SETBP1 mutations was conducted with several clinical factors adjusted; and the association was still significant (HR = 0.56, *P* = 0.002; Figure 2B). We also investigated the ICI prognostic roles of SETBP1 mutations in each melanoma cohort and the distinct therapeutic types used in this study (Supplementary Figures 3, 4, respectively). Further analyses revealed that a significant immunotherapeutic response advantage was found in the SETBP1-MUT subgroup (42.9% vs. 29.1%, Fisher exact test *P* = 0.016; Figure 2C). A multivariate logistic adjusted analysis consistently confirmed this result (OR = 0.61, *P* = 0.048; Figure 2D).

### Melanoma mutational burden according to SETBP1 mutational status

**Figure 3:**
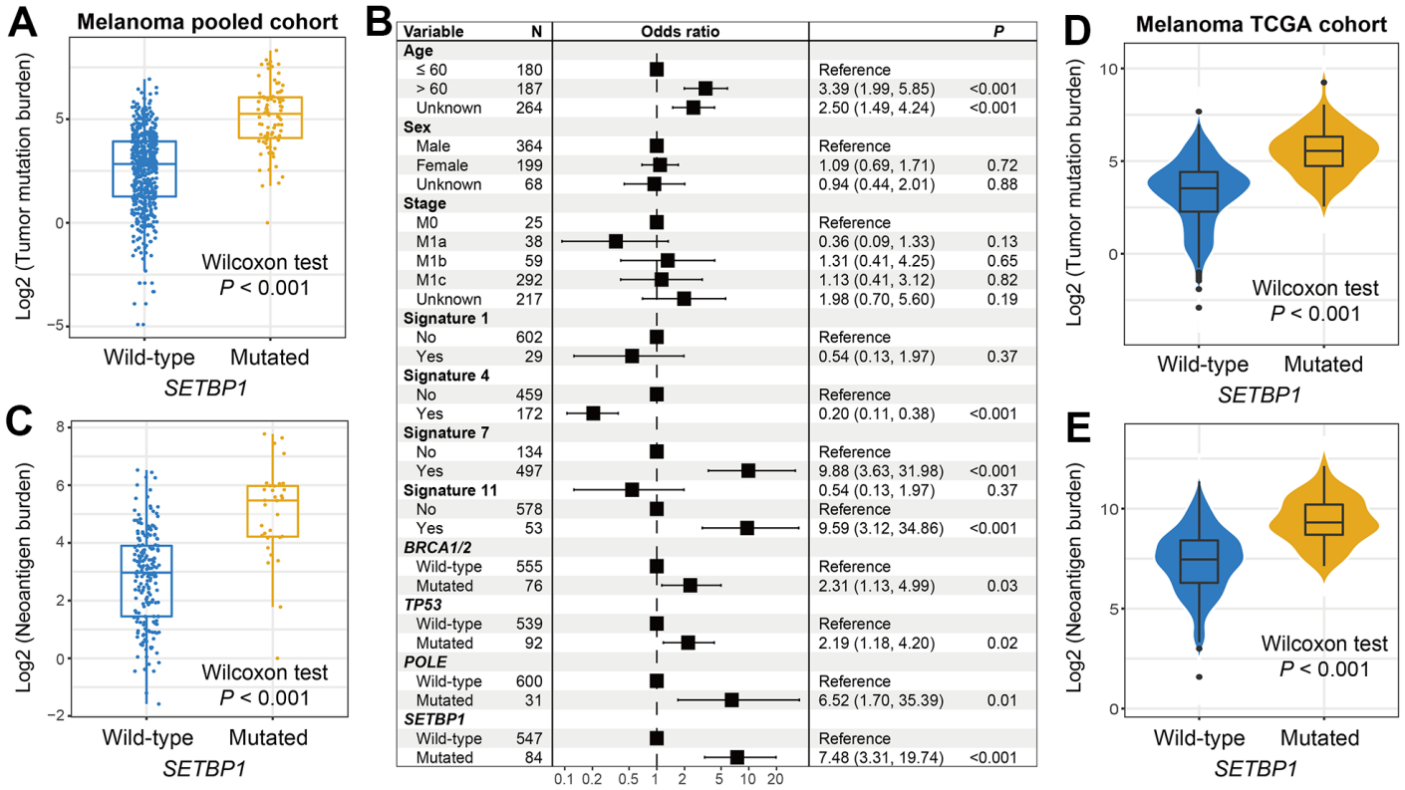
**Melanoma mutational burden according to SETBP1 mutational status.** (**A**) TMB level differences between SETBP1 mutated and wild-type subgroups. (**B**) A multivariate logistic regression analysis was performed to confirm the connection between SETBP1 mutations and TMB. (**C**) NB level differences of SETBP1 two subgroups. (**D**) TMB and (**E**) NB level differences between SETBP1 two subgroups based on the data from the TCGA cohort.

Tumor mutational burden (TMB) is recently reported as a hopeful indicator for cancer immunotherapies and its level is linked with tumor immunogenicity. We thus analyzed the connection of SETBP1 mutations with TMB in melanoma. Results showed that SETBP1-MUT patients harbored a significantly increased TMB than other patients (Wilcoxon rank-sum test *P* < 0.001; Figure 3A). Taking into account that tumor mutational signatures are deeply associated with the genomic mutational burden. We therefore extracted a total of 4 mutational signatures from melanoma mutational profiles (Supplementary Table 1). Subsequently, in a multivariate-adjusted model, we incorporated clinical confounders, identified mutational signatures, and genome maintenance gene alterations to elucidate the actual link between SETBP1 mutations and higher TMB. Consistently, the result was still significant (OR: 7.48, *P* < 0.001; Figure 3B). Moreover, an increased neoantigen burden (NB) was also enriched in the SETBP1-MUT group (Wilcoxon rank-sum test *P* < 0.001; Figure 3C). To further validate the above connections, we employed genomic and clinical data of melanoma samples from the Cancer Genome Atlas (TCGA). Expectantly, the significantly increased TMB and NB were both observed in the SETBP1-MUT patients (both *P* < 0.001; Figure 3D, 3E).

### SETBP1 mutations determined the sensitivity to ICI treatments in NSCLC

**Figure 4:**
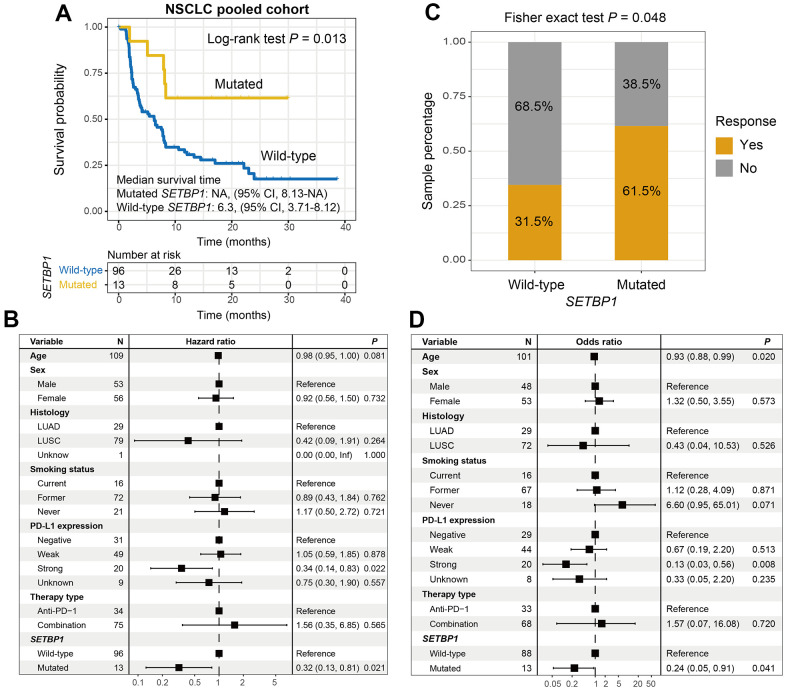
**SETBP1 mutations determined the sensitivity to ICI treatments in NSCLC.** (**A**) ICI survival differences between SETBP1 mutated and wild-type subgroups. (**B**) A multivariate Cox regression analysis was performed to verify the connection between SETBP1 mutations and ICI prognosis. (**C**) ICI response rate differences of SETBP1 two subgroups. (**D**) A multivariate logistic regression analysis was performed to verify the connection between SETBP1 mutations and ICI response rate.

Among the aggregated NSCLC samples, a total of 13 (11.9%) of 109 had SETBP1 mutations. Kaplan-Meier results demonstrated that SETBP1-MUT NSCLC patients presented a markedly improved ICI survival benefit than wild-type patients (median survival time: NA vs. 6.3 months, Log-rank test *P* = 0.013; Figure 4A). A multivariate Cox regression analysis of SETBP1 mutations was conducted with multiple clinical factors adjusted; and the connection still reached the statistical significance (HR = 0.32, *P* = 0.021; Figure 4B). We also explored the ICI prognostic roles of SETBP1 mutations in each NSCLC cohort and the distinct therapeutic types utilized in this study (Supplementary Figure 5). Further calculation indicated that an immunotherapeutic response advantage was noticed in such SETBP1-MUT group (61.5% vs. 31.5%, Fisher exact test *P* = 0.048; Figure 4C). A multivariate logistic adjusted analysis consistently validated this association (OR = 0.24, *P* = 0.041; Figure 4D).

**Figure 5:**
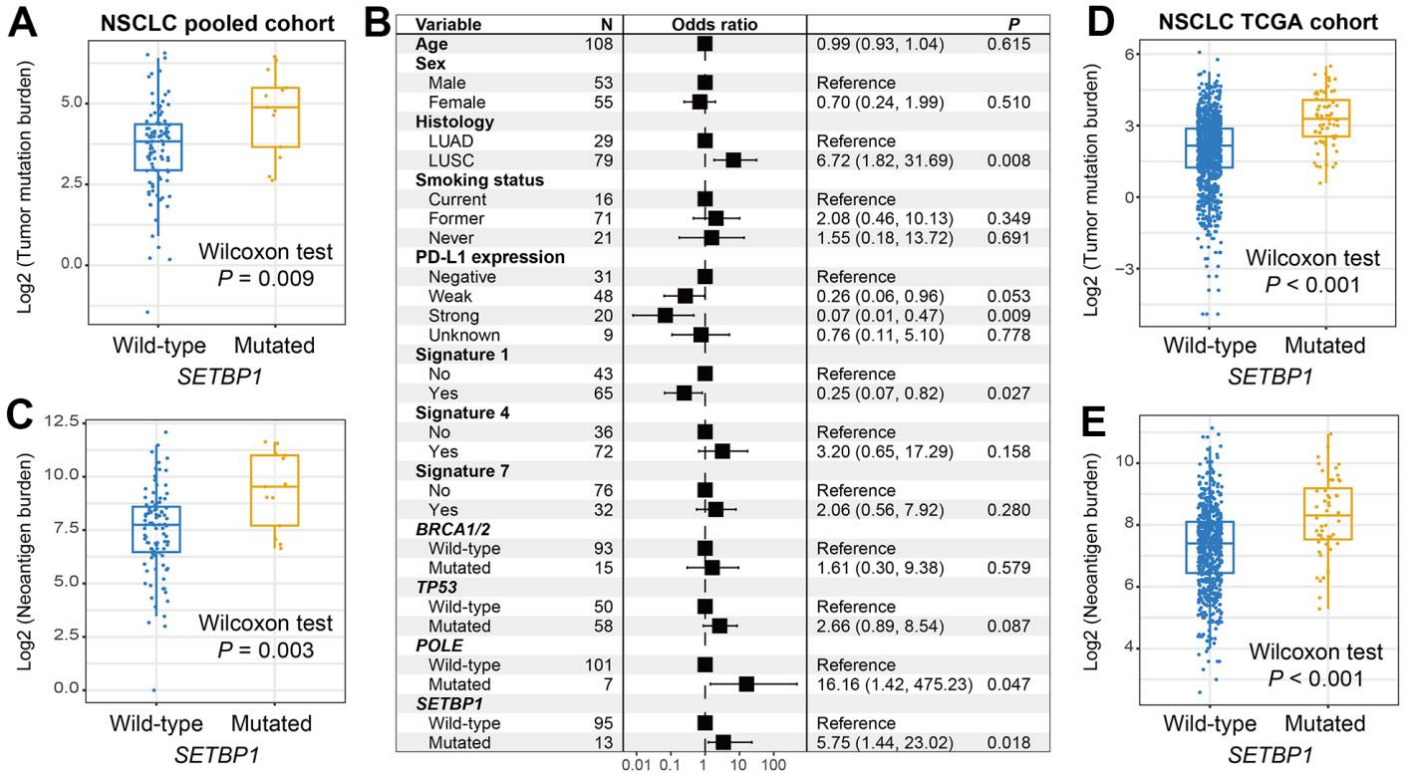
**NSCLC mutational burden according to SETBP1 mutational status.** (**A**) TMB level differences between SETBP1 mutated and wild-type subgroups. (**B**) A multivariate logistic regression analysis was performed to confirmed the connection between SETBP1 mutations and TMB. (**C**) NB level differences of SETBP1 two subgroups. (**D**) TMB and (**E**) NB level differences between SETBP1 two subgroups based on the data from the TCGA cohort.

Genomic mutational burden analysis showed that SETBP1-MUT NSCLC patients harbored a significantly increased TMB (Wilcoxon rank-sum test *P* = 0.009; Figure 5A). A total of 3 mutational signatures were determined from NSCLC mutational profiles (Supplementary Table 2). In a multivariate-adjusted analysis, we incorporated clinical confounders, identified mutational signatures, and genome maintenance gene mutations to investigate the actual connection between SETBP1 mutations and elevated TMB. Consistently, the connection was still meaningful (OR: 5.75, *P* = 0.018; Figure 5B). Furthermore, an increased NB was also noticed in this SETBP1-MUT subgroup (Wilcoxon rank-sum test *P* = 0.003; Figure 5C). Based on the genomic and clinical data of NSCLC samples from the TCGA, SETBP1-MUT patients also exhibited significantly enhanced TMB and NB (both *P* < 0.001; Figure 5D, 5E).

### Immunological properties behind SETBP1 mutations

**Figure 6:**
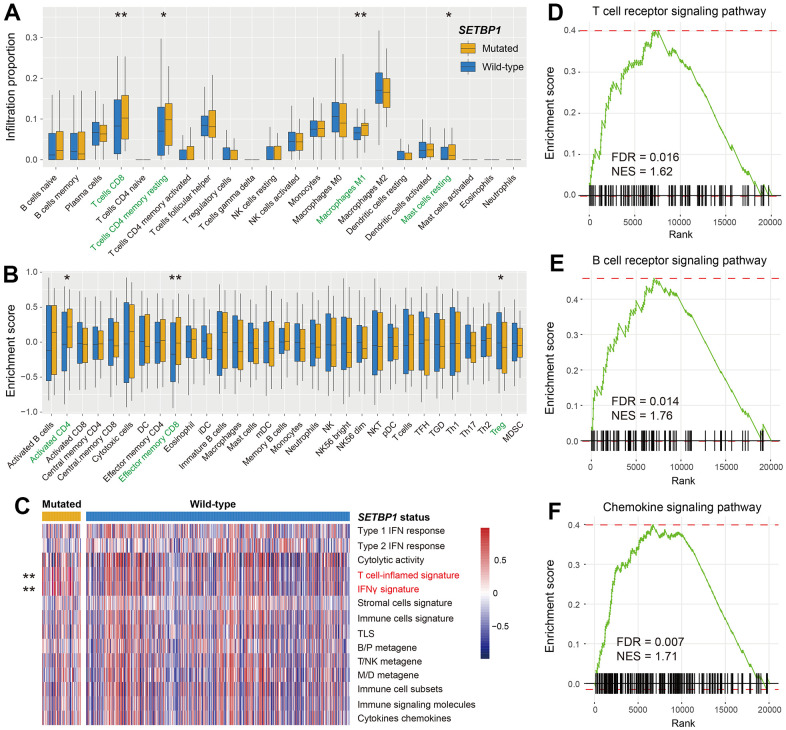
**Immunological implications behind SETBP1 mutations in melanoma.** (**A**) CIBERSORT algorithm revealed the 22 lymphocyte infiltration differences between SETBP1 mutated and wild-type subgroups. (**B**) Angelova et al. method revealed the 31 lymphocyte infiltration differences between SETBP1 two subgroups. (**C**) Heatmap illustration of enrichment scores of 14 immune-related molecular signatures according to SETBP1 mutational status. Signaling pathways of (**D**) T cell receptor signaling pathway, (**E**) B cell receptor signaling pathway, and (**F**) chemokine signaling pathway were enriched in SETBP1-MUT melanoma patients. * *P* < 0.05, ** *P* < 0.01.

The aforementioned results have indicated that SETBP1 mutations determined the ICI treatment efficacy. Therefore, we further explored the potential immunological properties of SETBP1 mutations. In melanoma, based on the lymphocyte infiltration methods, we observed more infiltration of immune-promotion cells (e.g., CD8 T cells and M1 macrophages) and less infiltration of immune-suppressive cells (e.g., regulatory T cells) in the SETBP1-MUT subgroup (all *P* < 0.05; Figure 6A, 6B). Subsequently, a series of tumor immunogenicity and immunotherapeutic response-relevant molecular signatures were presented with a heatmap according to SETBP1 mutational status (Figure 6C). Results revealed that the T cell-inflamed signature and IFNγ signature, which were previously reported to be predictive of a favorable anti-PD-1 therapeutic response [21], were significantly enriched in the SETBP1-MUT patients (both *P* < 0.01). Moreover, gene set enrichment analysis showed that pro-inflammatory related T cell receptor signaling pathway (FDR = 0.016; Figure 6D), B cell receptor signaling pathway (FDR = 0.014; Figure 6E), and chemokine signaling pathway (FDR = 0.007; Figure 6F) were all noticed in melanoma patients with SETBP1 mutations (Supplementary Figure 6).

We also performed immune infiltration evaluation with CIBERSORT and Angelova et al. methods for NSCLC patients. Results demonstrated that pro-inflammatory lymphocytes represented by CD8 T cells were significantly enriched in NSCLC patients with SETBP1 mutations (all *P* < 0.05; Supplementary Figure 7A, 7B).

## DISCUSSION

SETBP1 acts as a transcription factor in biological mechanisms and involves in tumor progression and immune regulation. ICI agents have exhibited remarkable therapeutic efficacy in tumor clinical practice. Nevertheless, most patients could not respond to such treatments. Therefore, novel immunotherapeutic biomarkers are necessary. In this work, we determined that SETBP1 mutations were connected with genomic mutational burden, tumor immunogenicity, and importantly, ICI treatment sensitivity. The observations gleaned from this study may provide a new try for selecting cancer ICI biomarkers.

In cancers, familiar biological functions for SETBP1 are related to DNA replication. Recently, immune regulation roles have also been revealed by several studies. Carratt et al. reported that mutant SETBP1 promoted the activity of the MYC pathway [15], which is involved in innate immunity [22], antiviral immune response [23], and immune escape [24]. Activation of the MAPK pathway was demonstrated to associate with the cancer immune microenvironment [25], inflammatory reprogramming [26], and immune regulation [27]. Mutated SETBP1 also enhanced the capacity of the MAPK signal to facilitate tumor progression [16]. The above observations further confirm the immunological implications of SETBP1 mutations and support the results derived from our study.

To our knowledge, TMB is a promising determinant for cancer immunotherapeutic response [28, 29]. We also detected the distinct distribution of TMB in SETBP1 two groups. Results showed that the SETBP1-MUT group exhibited an elevated TMB and this finding partially explains why SETBP1-MUT patients harbored a preferable ICI efficacy. Furthermore, we conducted an evaluation of the TMB distribution in distinct SETBP1 mutational statuses among melanoma and NSCLC cell lines derived from the CCLE project. Our analysis encompassed 57 melanoma cell lines and 98 NSCLC cell lines. We consistently observed strong associations between SETBP1 mutations and increased TMB in both tumors (Wilcoxon rank-sum test *P* = 0.028 and 0.013 for melanoma and NSCLC, respectively; Supplementary Figure 8A, 8B). Despite the powerful capacity of TMB, its accurate evaluation needs to perform whole-exome sequencing. And uncertain cut-off values for TMB in distinct cancer types are another reason for limiting its wide application [28]. Based on the evidence from our study, sequencing only SETBP1 mutation may be a surrogate for TMB to evaluate immunotherapeutic sensitivities.

In order to examine the possible immune infiltration and immune signature enrichment associated with SETBP1 mutations, we utilized two immune infiltration methods and collected data on 14 immune-related signatures. Our results revealed that patients with SETBP1 mutations demonstrated significantly elevated levels of CD8 T cell infiltration and greater enrichment of IFNγ signature. Previous research has indicated a positive correlation between CD8 T cells and IFNγ production. The favorable tumor immunogenicity of SETBP1 mutations may explain the enhanced efficacy of ICI treatment.

We investigated whether SETBP1 mutations mediate immune regulation and ICI treatment efficacy by regulating their own expression. Results from the CCLE cell lines showed a tendency of decreased SETBP1 expression in the SETBP1 mutant group in both melanoma and NSCLC, although it did not reach statistical significance (Wilcoxon rank-sum test *P* = 0.068 and 0.153 for melanoma and NSCLC, respectively; Supplementary Figure 9A, 9B). Further analysis based on samples from the TCGA cohort revealed significantly reduced SETBP1 expression in the SETBP1 mutant subgroup in both melanoma (Wilcoxon rank-sum test *P* = 0.034; Supplementary Figure 9C) and NSCLC (Wilcoxon rank-sum test *P* = 0.008; Supplementary Figure 9D). These findings suggest that SETBP1 mutations may regulate their own expression to modulate immune functions and treatment efficacy in the context of ICI therapy.

Previously several studies have revealed that SETBP1 mutations were associated with poor survival outcomes in cancers [15–17]. We further investigated the role of SETBP1 mutations in melanoma and NSCLC patients who received conventional chemotherapies from the TCGA. Survival analysis indicated that there was no significant survival difference between SETBP1 two groups (multivariate Cox *P* = 0.143; Supplementary Figure 10A) in melanoma; however, a significantly preferable prognosis was observed in SETBP1-MUT NSCLC patients (multivariate Cox *P* = 0.043; Supplementary Figure 10B). In this work, SETBP1 mutations were also identified to be linked with favorable ICI treatment outcomes in both melanoma and NSCLC. The above findings suggest that SETBP1 mutations may be a predictive biomarker for cancer immunotherapies or chemotherapies, rather than a prognostic biomarker.

During oncogenesis, neoplastic cells not only evade the body’s regulatory mechanisms but also acquire the ability to perturb local and systemic homeostasis. Specifically, tumors produce a variety of molecules including cytokines, immune mediators, classical neurotransmitters, hypothalamic and pituitary hormones, biogenic amines, melatonin, and glucocorticoids, as demonstrated by human and animal models of cancer [30]. Through the release of these neurohormonal and immune mediators, tumors can manipulate the major neuroendocrine centers such as the hypothalamus, pituitary, adrenals, and thyroid, to regulate body homeostasis via central regulatory axes [30].

There are several limitations to our study. First, all relevant results were obtained based on the *in-silico* analysis, no in-depth experimental validations were performed. Second, the ICI-treated melanoma and NSCLC cohorts were integrated from multiple smaller datasets, which may bring some data biases. Third, only two cancer types were employed in assessing the immunotherapeutic efficacy of SETBP1 mutations, no additional cancers with both mutational profiles and ICI treatment information were acquired. Final, it is well-established that the melanin pigment pathway plays a critical role in melanoma progression and occurrence, and can significantly impact tumor behavior, immune responses, and therapeutic efficacy [31]. Nevertheless, due to the absence of this data in our research, we could not perform a more in-depth analysis. Therefore, future studies that include relevant data are necessary to gain a deeper understanding of this pathway’s role in cancer development and treatment.

In summary, by using clinically expanded ICI cohorts, we uncovered that SETBP1 mutations were associated with favorable tumor immunogenicity and determined the sensitivity to cancer immunotherapies, which provides a potential biomarker for evaluating cancer ICI treatment response.

## MATERIALS AND METHODS

### Collection of samples

We comprehensively searched previously published studies and cancer-related databases to obtain cancer patients with both genomic mutational data and clinical ICI treatment information. Finally, a total of 631 melanoma [32–39] and 109 NSCLC samples [40, 41] reached the inclusion criteria and were employed in this study. All included samples were treated with ICI agents of anti-PD-1/PD-L1, anti-CTLA-4, or a combination. Detailed clinical characteristics for melanoma and NSCLC samples were illustrated in Supplementary Tables 3, 4, respectively. Taking into account that original mutation data were sequenced from distinct platforms, we therefore used the mutation annotation software of Oncotator to uniformly annotate them. For molecular mechanistic analysis and specific prognosis validation, we also acquired melanoma and NSCLC samples with transcriptomic and mutational data from the TCGA project (http://xena.ucsc.edu/).

In the cellular level, we obtained a total of 57 cell lines for the melanoma and 98 cell lines for the NSCLC with both somatic mutational data and transcriptomic mRNA expression profiles from the Cancer Cell Line Encyclopedia (CCLE) project (https://depmap.org/portal/ccle/) to validate the relevant connections.

### Tumor infiltration lymphocytes

CIBERSORT algorithm [42] was utilized to calculate the distinct infiltration abundance of 22 lymphocytes between SETBP1 mutated and wild-type subgroups. Besides, Angelova et al. evaluated the infiltration levels of 31 lymphocyte subtypes [43] by using 812 feature genes (Supplementary Table 5). In this study, the above two methods were used to obtain comprehensive results.

### Tumor immunogenicity and immunotherapeutic response-related signatures

Recently multiple studies have reported the molecular signatures associated with tumor immunogenicity and ICI treatment sensitivity. We curated a total of 14 signatures with detailed feature genes in Supplementary Table 6.

### Enrichment of dysregulated signaling pathways

According to the SETBP1 mutational status, we partitioned included melanoma or NSCLC samples into two groups. Subsequently, transcriptomic differential analyses of the whole genome of divided two groups were performed by employing the DESeq2 R package [44]. All *t* values obtained from the differential analysis result were used to conduct gene set enrichment analysis (GSEA). Signaling pathways stored in the KEGG database were regarded as the background reference. In addition, to evaluate the detailed enrichment scores of collected Angelova et al. lymphocytes and immune-related signatures, a single sample GSEA (within the R GSVA package [45]) was used based on their corresponding representative genes.

### Statistical analysis

R software (version 4.2.1) was used in this study to achieve related analyses and figures. Tumor mutational signatures were determined according to a procedure reported by Kim et al. [46]. Waterfall plot was utilized to show mutational features of driver genes under the maftools package [47]. Heatmap illustration of molecular signatures in SETBP1 two subgroups was achieved using the pheatmap package. Wilcoxon rank-sum test (Wilcoxon test) and Fisher exact test were performed to respectively calculate the connection of continuous and categorical variables with SETBP1 statuses.

### Data availability statement

All data used in this study are acquired from publicly available cohorts.

## Supplementary Material

Supplementary Figures

Supplementary Table 1

Supplementary Table 2

Supplementary Table 3

Supplementary Table 4

Supplementary Table 5

Supplementary Table 6
