# Supplementary material for: SETBP1 mutation determines sensitivity to immune checkpoint inhibitors in melanoma and NSCLC
Source: Aging (Albany NY). 2023 Aug 2;15(15):7476–95. doi: 10.18632/aging.204913 (PMC10457048; doi:10.18632/aging.204913)
Supplement: Supplementary Figures [file aging-15-204913-s001.pdf]

SUPPLEMENTARY FIGURES

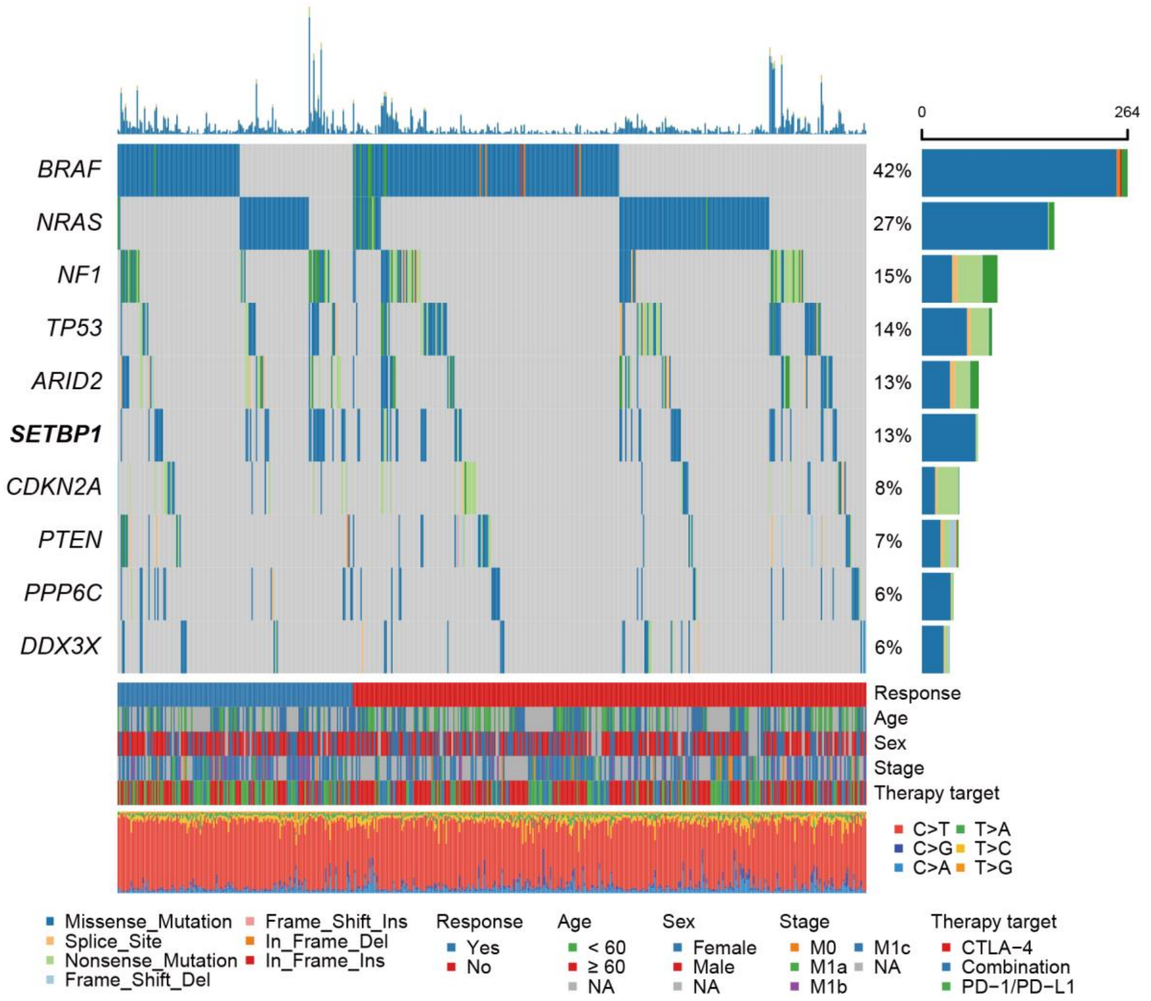

Supplementary Figure 1. Mutational patterns of SETBP1 and common melanoma driver genes illustrated with waterfall plot.

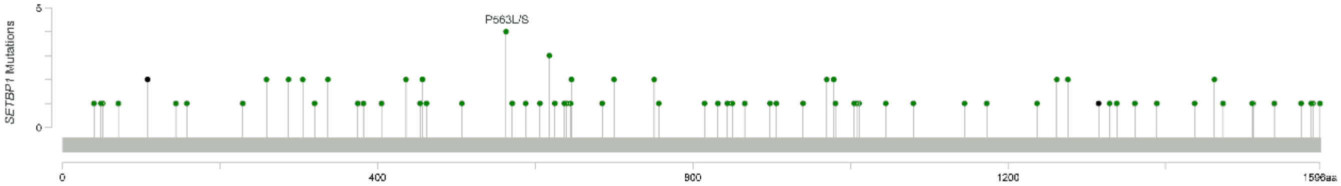

Supplementary Figure 2. Detailed amino acid changes induced by SETBP1 mutations in the integrated melanoma cohort.

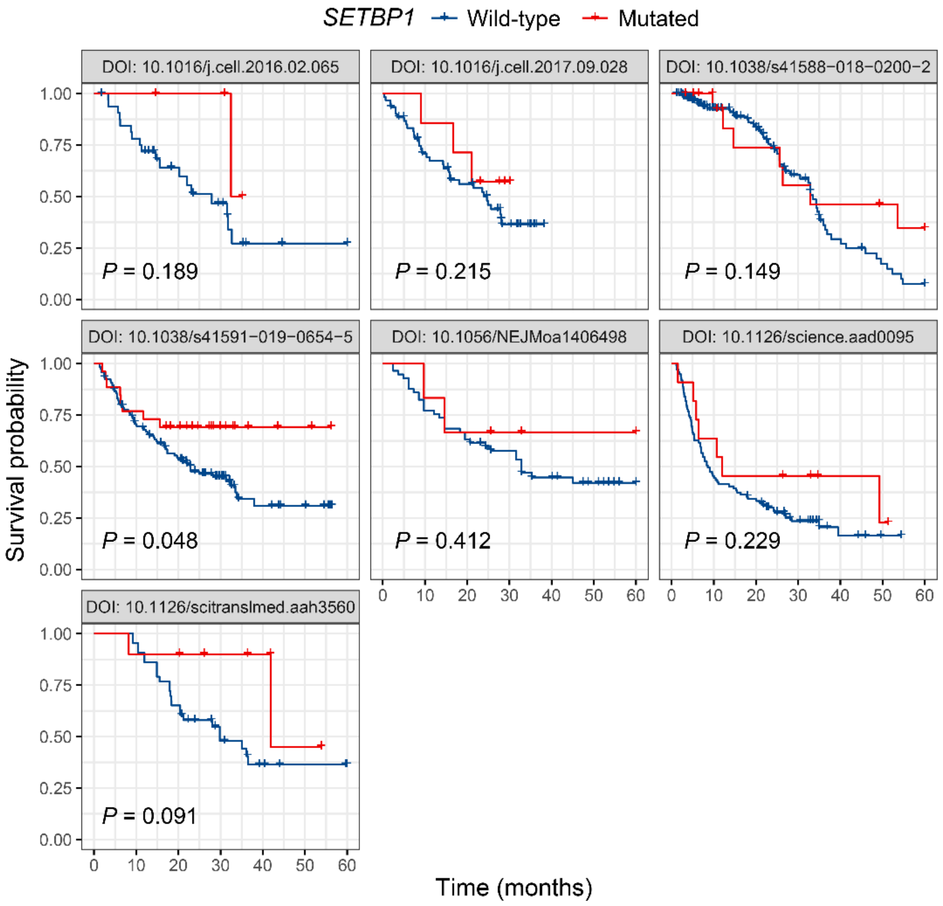

Supplementary Figure 3. Kaplan-Meier survival analyses of SETBP1 mutations in individual ICI-treated melanoma cohorts.

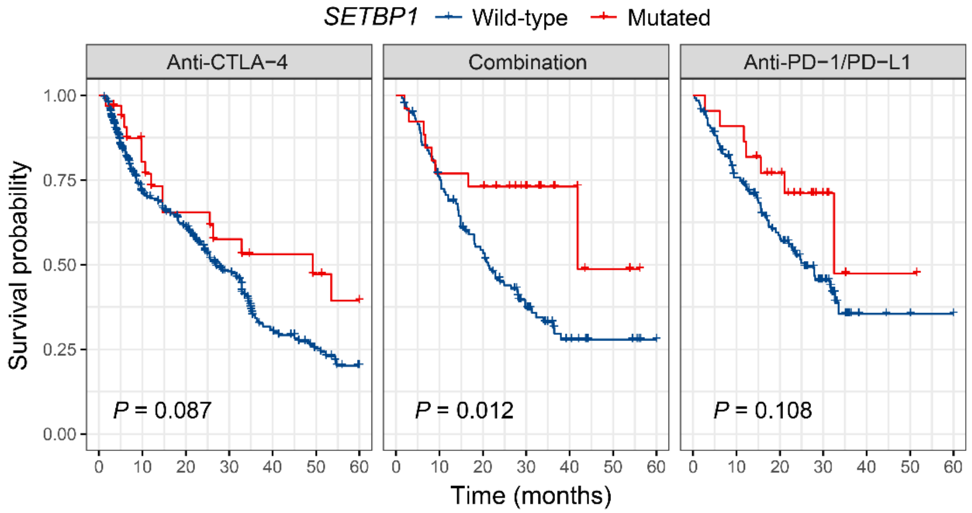

Supplementary Figure 4. Kaplan-Meier survival analyses of SETBP1 mutations in distinct ICI treatment types in melanoma.

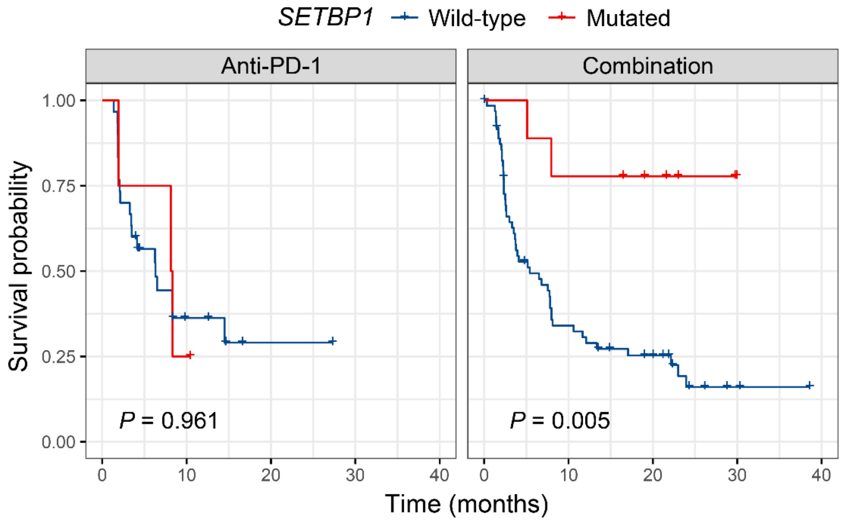

Supplementary Figure 5. Kaplan-Meier survival analyses of SETBP1 mutations in individual ICI-treated NSCLC cohorts.

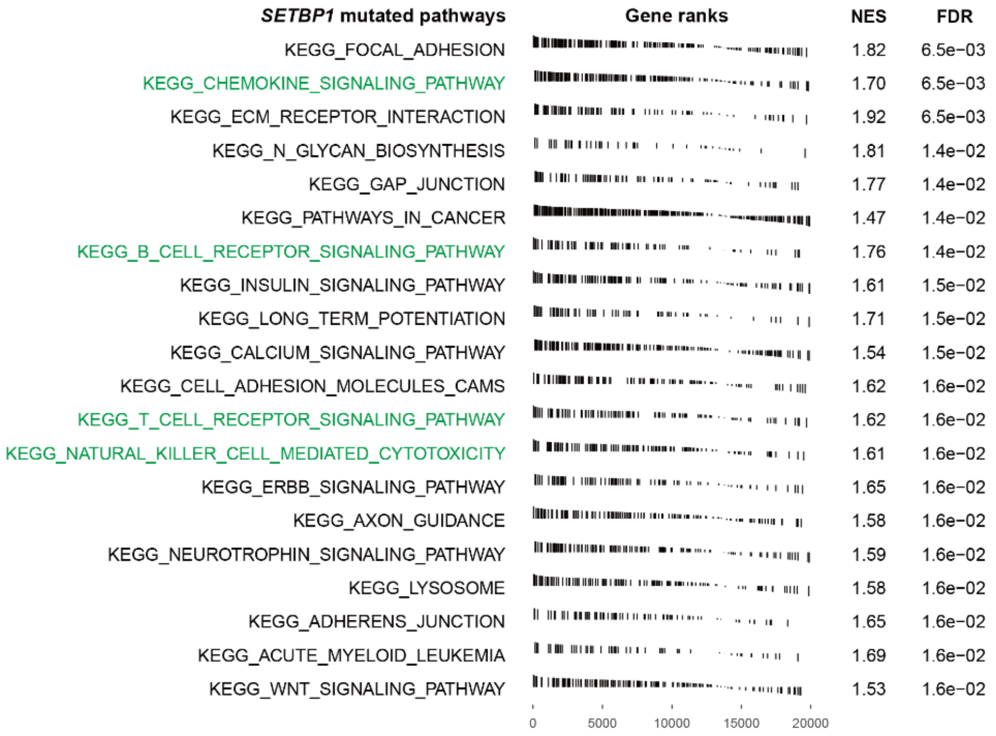

Supplementary Figure 6. Significantly enriched signaling pathways in SETBP1 mutated subgroups in melanoma. Immune response pathways were highlighted with green.

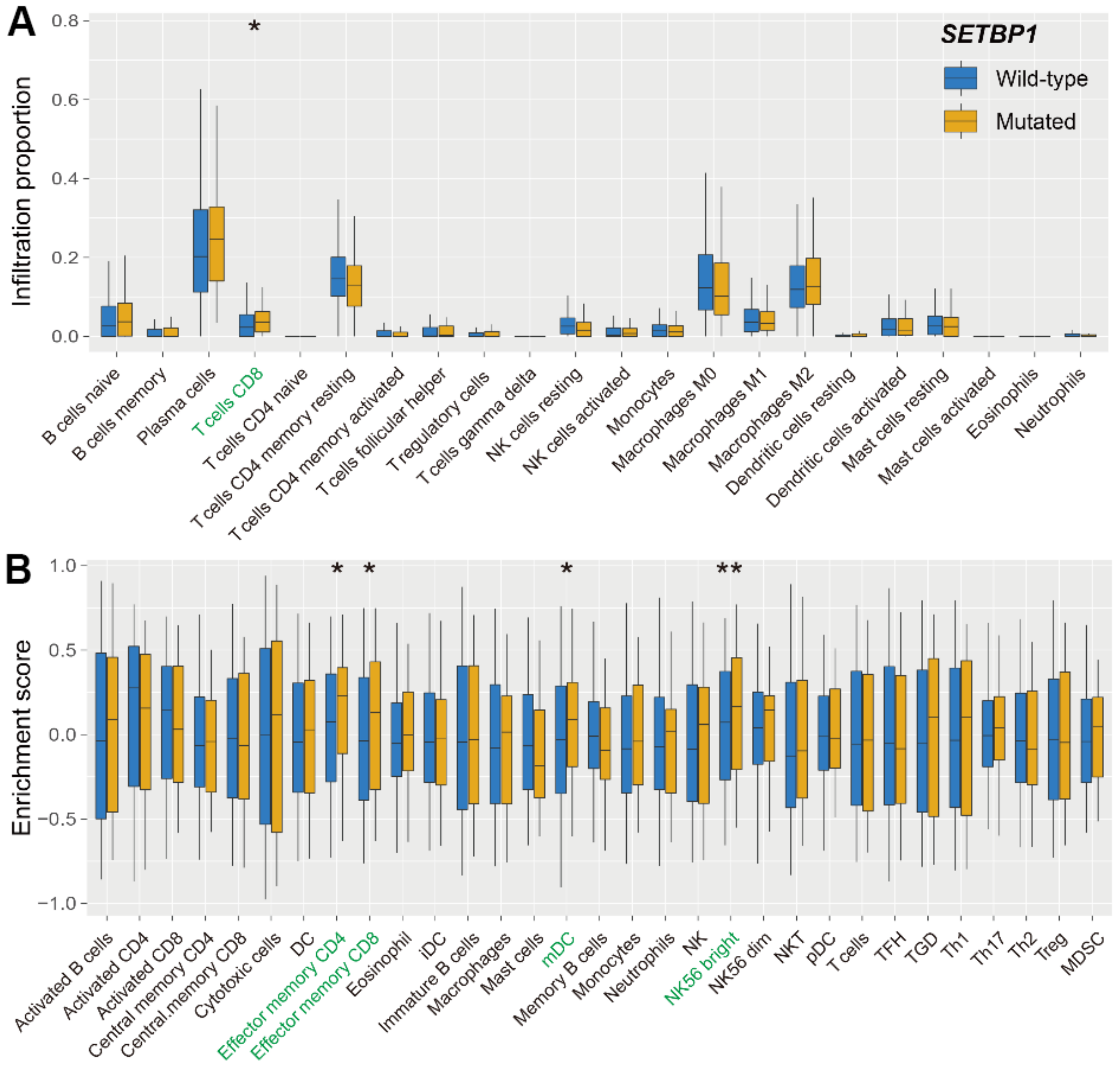

Supplementary Figure 7. Immune infiltration associated with SETBP1 mutations in NSCLC. (**A**) Distinct infiltration of 22 immunocytes of SETBP1 mutated and wild-type groups evaluated with CIBERSORT algorithm. Immunocytes highlighted with green are significantly differentially infiltrated. (**B**) Distinct infiltration of 31 immunocytes of SETBP1 two groups evaluated with Angelova et al. method.

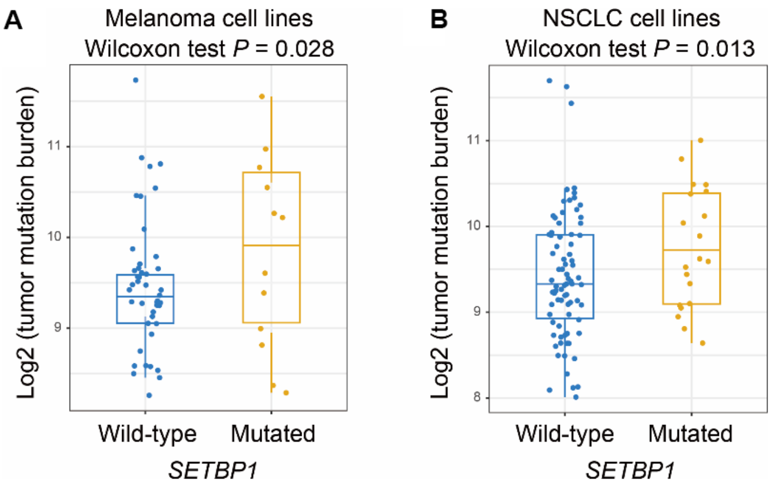

Supplementary Figure 8. Associations between SETBP1 mutations and TMB in CCLE-derived cell lines for melanoma and NSCLC. (**A**) Distinct TMB distribution in SETBP1 mutated and wild-type subgroups based on 57 melanoma cell lines. (**B**) Distinct TMB distribution in SETBP1 mutated and wild-type subgroups based on 98 NSCLC cell lines.

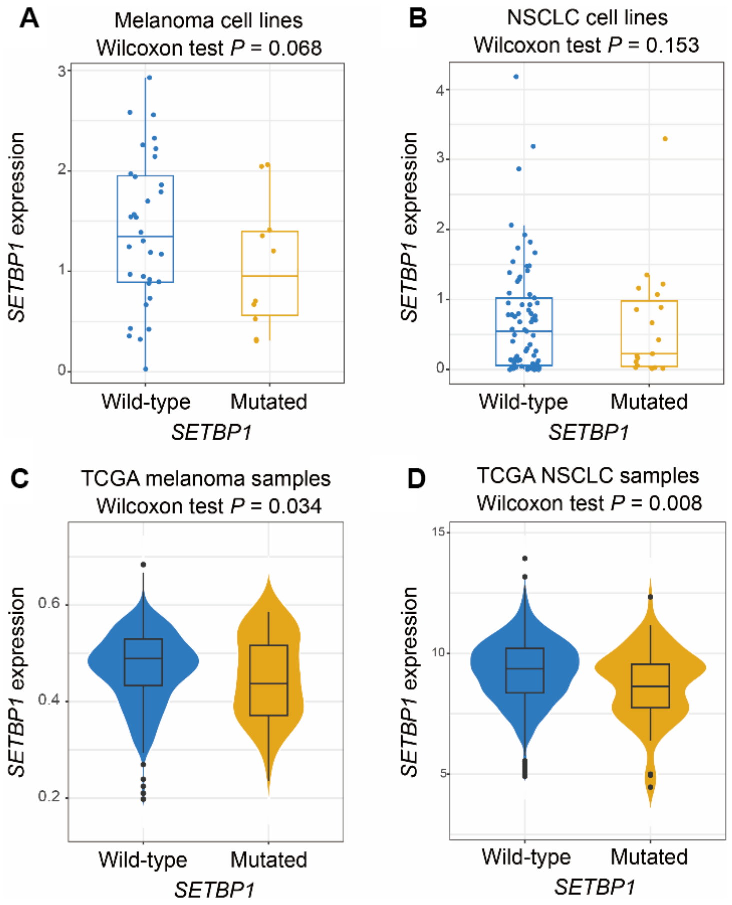

Supplementary Figure 9. Associations between SETBP1 mutations and its own expression. (**A**) Distinct SETBP1 expression in SETBP1 mutated and wild-type subgroups based on 42 melanoma cell lines. (**B**) Distinct SETBP1 expression in SETBP1 mutated and wild-type subgroups based on 92 NSCLC cell lines. (**C**) Distinct SETBP1 expression in SETBP1 mutated and wild-type subgroups based on TCGA melanoma samples. (**D**) Distinct SETBP1 expression in SETBP1 mutated and wild-type subgroups based on TCGA NSCLC samples.

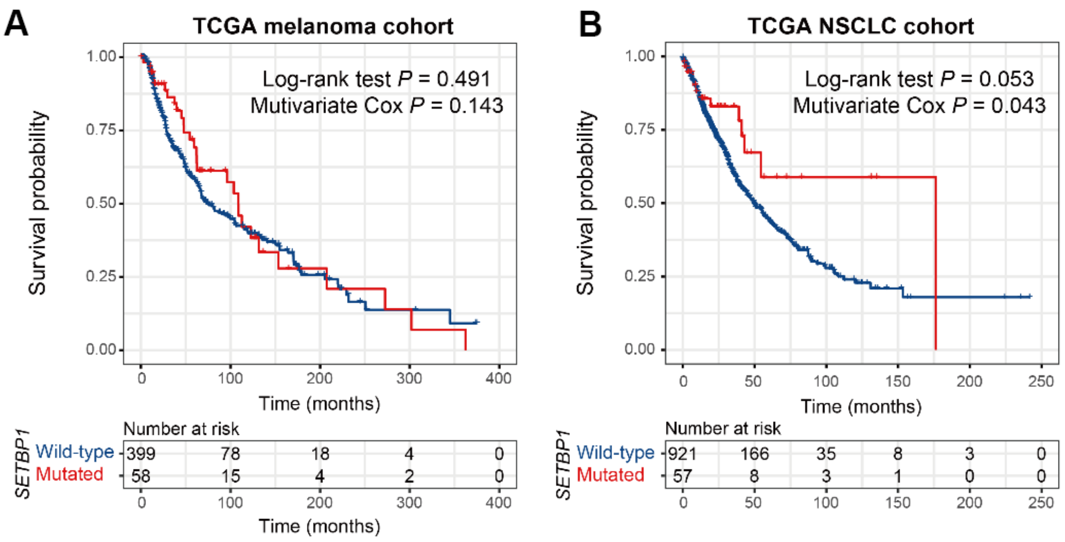

Supplementary Figure 10. Prognostic capacities of SETBP1 mutations in (**A**) melanoma and (**B**) NSCLC patients derived from the TCGA project.
